# Xylopic acid-amodiaquine and xylopic acid-artesunate combinations are effective in managing malaria in *Plasmodium berghei*-infected mice

**DOI:** 10.1186/s12936-021-03658-6

**Published:** 2021-02-25

**Authors:** Silas Acheampong Osei, Robert Peter Biney, Ernest Obese, Mary Atta-Panyi Agbenyeku, Isaac Yaw Attah, Elvis Ofori Ameyaw, Johnson Nyarko Boampong

**Affiliations:** 1 Department of Biomedical Sciences, School of Allied Health Sciences, University of Cape Coast, Cape Coast, Ghana; 2 School of Pharmacy and Pharmaceutical Sciences, University of Cape Coast, Cape Coast, Ghana; 3 Department of Pharmacology, School of Medical Sciences, University of Cape Coast, Cape Coast, Ghana

**Keywords:** Antimalarial drugs, Combination therapies, Isobolographic analysis, Xylopic acid, Artesunate, Amodiaquine, Synergism, *Plasmodium berghei*

## Abstract

**Background:**

Evidence of *Plasmodium* resistance to some of the current anti-malarial agents makes it imperative to search for newer and effective drugs to combat malaria. Therefore, this study evaluated whether the co-administrations of xylopic acid-amodiaquine and xylopic acid-artesunate combinations will produce a synergistic anti-malarial effect.

**Methods:**

Antiplasmodial effect of xylopic acid (XA: 3, 10, 30, 100, 150 mg kg^−1^), artesunate (ART: 1, 2, 4, 8, 16 mg kg^−1^), and amodiaquine (AQ: 1.25, 2.5, 5, 10, 20 mg kg^−1^) were evaluated in *Plasmodium berghei* (strain ANKA)-infected mice to determine respective ED_50_s. Artemether/lumefantrine was used as the positive control. XA/ART and XA/AQ were subsequently administered in a fixed-dose combination of their ED_50_s (1:1) and the combination fractions of their ED_50_s (1/2, 1/4, 1/8, 1/16, and 1/32) to determine the experimental ED_50_s (Z_exp_). An isobologram was constructed to determine the nature of the interaction between XA/ART, and XA/AQ combinations by comparing Z_exp_ with the theoretical ED_50_ (Z_add_). Bodyweight and 30-day survival post-treatment were additionally recorded.

**Results:**

ED_50_s for XA, ART, and AQ were 9.0 ± 3.2, 1.61 ± 0.6, and 3.1 ± 0.8 mg kg^−1^, respectively. The Z_add_, Z_exp,_ and interaction index for XA/ART co-administration was 5.3 ± 2.61, 1.98 ± 0.25, and 0.37, respectively while that of XA/AQ were 6.05 ± 2.0, 1.69 ± 0.42, and 0.28, respectively. The Z_exp_ for both combination therapies lay significantly (p < 0.001) below the additive isoboles showing XA acts synergistically with both ART and AQ in clearing the parasites. High doses of XA/ART combination significantly (p < 0.05) increased the survival days of infected mice with a mean hazard ratio of 0.40 while all the XA/AQ combination doses showed a significant (p < 0.05) increase in the survival days of infected mice with a mean hazard ratio of 0.27 similar to AL. Both XA/ART and XA/AQ combined treatments significantly (p < 0.05) reduced weight loss.

**Conclusion:**

Xylopic acid co-administration with either artesunate or amodiaquine produces a synergistic anti-plasmodial effect in mice infected with *P. berghei*.

## Background

Regardless of the efforts put in place in the twenty-first century to eradicate the staggering toll of malaria on human health, the global burden of the disease remains, especially, in several tropical countries. The World Health Organization (WHO) estimates that 40% of the world’s population is susceptible to malaria infections [1]. A recent report indicates that 228 million cases of malaria occurred in 2018, which resulted in 405,000 deaths, mostly in sub-Saharan Africa [2]. About 93% (213 million) of the cases in 2018 were recorded in the WHO African Region. Ghana and Nigeria are the two countries among the 10 highest-burden countries in Africa, which recorded an increase in malaria cases from 2017 to 2018. Children under five years succumb to the devastating effects of the disease, accounting for 272,000 (67%) of all malaria deaths worldwide [2]. The incidence rate and the death toll of malaria on children make the disease a major global infectious disease.

Cinchona alkaloids (quinine and quinidine) and artemisinin derivatives (artesunate, artemether, and arteether) are the two classes of medicines available for the treatment of severe and uncomplicated malaria. *Plasmodium falciparum* has developed resistance to anti-malarial agents, such as chloroquine in the past and there are reports of the growing resistance of *P. falciparum* to artemisinin derivatives in South-east Asia [3]. An anecdotal study in six West-African countries, including Ghana showed increased failure rates (10%) in malaria treatment with artemisinin-based combination therapy [4]. Some malaria vaccines (*Plasmodium falciparum* sporozoite vaccine (PfSPZ), Chemoprophylaxis vaccination (CVac), Genetically-attenuated parasite vaccine (GAP), RTS,S/AS01) are at various stages of development, but a clinically approved malaria vaccine is not available, therefore [5] making the search for newer, more effective anti-malarial agent still relevant.

Drug combination therapies (DCTs) are pertinent to the optimum control of malaria in developing countries [6] because they provide improved efficacy and might also give synergistic activity. Due to the rapid spread of drug resistance among parasites worldwide, the initial use of single drugs as monotherapies has given way in the last decades to combination therapies of two or more drugs especially the use of agents with different modes of action to improve efficacy and reduce resistance [7, 8]. Drug combinations also enhance the probability that one agent can be at least clinically active in the case of parasite resistance to the drug. For example, in East Africa, malaria parasites are resistant to both amodiaquine and sulfadoxine-pyrimethamine (SP), but the combination of these two agents still gives an excellent anti-malarial efficacy [9–11].

Natural products are essential in the drug discovery process, and there is no exception in anti-malarial agents. Medicinal plant extracts have been a source for anti-malarial drug discovery for long, and their treatment for malaria has been successful [12]. About 160 plant families have been established to have anti-malarial properties. From these families, more than 1200 species have been documented to have anti-malarial properties [1], including *Xylopia aethiopica* which is used to treat malaria by Ghanaian herbal practitioners [13].

Xylopic acid, a kaurene diterpene, is the major constituent of the fruits of *Xylopia aethiopica* and has been reported to possess anti-malarial properties in *Plasmodium berghei*-infected ICR mice. Furthermore, it significantly reduced the lipopolysaccharide—(LPS) induced fever in Sprague–Dawley rats [13]. Thus, xylopic acid possesses prophylactic and curative anti-malarial effects along with antipyretic and analgesic properties, making it a promising anti-malarial agent. Artesunate, amodiaquine, and xylopic acid have all been shown to be effective in combination therapies as demonstrated by Ameyaw et al. [14] on the synergistic effect of xylopic acid in combination with cryptolepine in clearing malaria parasites in a malaria experimental model. Similarly, the anti-malarial activity of amodiaquine and artesunate was enhanced when combined with lopinavir/ritonavir [15]. In the present study, we tested the efficacy of xylopic acid/amodiaquine and xylopic acid/artesunate combination therapy in mice infected with *P. berghei*.

## Methods

### Xylopic acid extraction

Xylopic acid was extracted from *Xylopia aethiopica* as previously described [16–18]. Fresh unripe fruits of *X. aethiopica* purchased from the Ho Central Market in Ghana were shade-dried and pulverized with a hammer mill. For every 100 g of plant material, 300 ml of petroleum ether was used as a solvent for maceration. The mixture of *X. aethiopica* and petroleum ether was left to stand for 3 days with continuous shaking every 24 h. Whatman filter paper was used to filter the mixture and left to stand overnight under dark conditions. The filtrate was then concentrated with a Rotary evaporator (Heidolph Labo Rota, 4002) at 120 revolutions per minute and 40–55 °C. The concentrate was left was to stand for 72 h and 3 drops of ethyl acetate added to facilitate the crystallization of crude xylopic acid crystals. Crude xylopic acid was washed several times with petroleum ether and dissolved in absolute ethanol for purification by recrystallization. The purity of the xylopic acid was assessed by thin-layer chromatography using petroleum ether and ethyl acetate (9:1) as the solvent system. Pure xylopic acid was used as a reference and both compounds gave Rf of 0.53.

### Experimental animals

Six to ten weeks-old female ICR mice purchased from the Centre for Medicinal Plant Research, Akuapim-Mampong, Ghana, were used for the study. They were housed in stainless steel cages (16.5 × 11.0 × 13.5 cm^3^) with beddings made from softwood shavings. The animals were kept under appropriate laboratory conditions and fed with a normal commercial pellet diet purchased from Agricare, (Kumasi, Ghana) and water ad libitum. The cages were kept in the Department of Biomedical Sciences animal holding facility, University of Cape Coast, and the wood shavings were replaced every 3 days and disinfected with 70% alcohol. The facility had a 12/12 h light/dark cycles and a mean temperature of 21 °C.

### Drugs, chemicals, and reagents

Artemether/lumefantrine combined tablets (20/120 mg), artesunate, and amodiaquine were acquired from Novartis Pharma AG Basel, Switzerland. Hydrochloric acid, Giemsa stain, absolute methanol, chloroform, petroleum ether, ammonium hydroxide, 96% ethanol, liquid paraffin, Tween 20, and ammonium chloride were also purchased from Sigma Aldrich. St Louis, MO, USA.

### *Plasmodium berghei* ANKA parasite acquisition and inoculation

A chloroquine-sensitive strain of rodent malaria parasite *Plasmodium berghei* (strain ANKA) was acquired from Noguchi Memorial Institute for Medical Research (NMIMR), University of Ghana and by a continuous passage in mice intraperitoneally every 6 days [19]. Once high parasitaemia (30–40%) was established in a donor mouse, it was sedated under chloroform, following Hoff’s technique [20]. Blood was collected by cardiac puncture and transferred into EDTA tubes, capped, and topped with Phosphate Buffered Saline (PBS). The mixture containing blood, EDTA, and PBS were washed three times by centrifuging with a haematocrit centrifuge, at 15,000 rpm for 6–7 min to obtain pellets. Total inoculum concentration of 17.4 × 10^7^ *P. berghei* parasitized erythrocytes were prepared and each mouse was inoculated with 0.20 ml of PBS containing 1.2 × 10^6^ parasitized red blood cells.

### Bodyweight measurement

Mice body weights were measured on days 0 and day 7 post-infection following a procedure described by Dikasso and colleagues [21] using a top pan balance (Toledo® Metler, Japan).

### Anti-malarial activity

#### In vivo* anti-plasmodial assay of xylopic acid, artesunate, and amodiaquine monotherapies*

To confirm the reported anti-malarial properties of xylopic acid (XA), artesunate (ART), and amodiaquine (AQ), and also determine their ED_50_ values for the isobolographic analysis, the anti-plasmodial activity of each compound was assessed. After infection, mice were assigned to 18 groups (n = 5). Mice in all groups were inoculated with *P. berghei* except Group 18 mice which served as naïve control. Seventy-two hours post-inoculation (day 3), all groups of animals were treated once daily by oral administration with a gastric gavage with either xylopic acid (3, 10, 30, 100, 150 mg kg^−1^), artesunate (1, 2, 4, 8, 16 mg kg^−1^), amodiaquine (1.25, 2.5, 5, 10, 20 mg kg^−1^), artemether/lumefantrine (1.14/6.9 mg kg^−1^), or vehicle, 10 ml kg^−1^ (naïve and sham control). The ED_50_ values obtained as fitted midpoints of XA, ART and AQ were determined by iterative curve fitting of log-dose responses of XA, ART, and AQ. Mice were observed at 12 h intervals for death and the median survival and hazard ratio over a 30-day period was computed.

#### In vivo isobolographic assessment of xylopic acid-artesunate co-administration on *P. berghei*-induced malaria

To assess the antiplasmodial property of xylopic acid-artesunate (XA/ART) co-administration on established *P. berghei* infection, 6 to 10 weeks female ICR mice were each inoculated with 1.2 × 10^6^ in 0.20 ml PBS and assigned to 8 groups (n = 5). On day 3, each group received either fixed ratio (1:1) of the ED_50s_ of XA and ART (9 + 1.6 mg kg^−1^) or combinations of fractions of the respective ED_50_ values: ED_50_ (XA/ART)/2, (4.5 + 0.8 mg kg^−1^), ED_50_ (XA/ART)/4 (2.25 + 0.4 mg kg^−1^), ED_50_ (XA/ART)/8, (1.13 + 0.2 mg kg^−1^), ED_50_ (XA/ART)/16 (0.6 + 0.1 mg kg^−1^. Positive control (AL) and negative control (sham) mice received 1.14/6.9 mg kg^−1^ AL and 10 ml kg^−1^ vehicle, respectively.

#### In vivo isobolographic assessment of xylopic acid-amodiaquine co-administration on *P. berghei*-induced malaria

To assess the anti-plasmodial property of xylopic acid-amodiaquine (XA/AQ co-administration, on established *P. berghei* infection, 6 to 10 weeks female ICR mice were each inoculated with 1.2 × 10^6^ in 0.2 ml PBS and assigned to 8 groups (n = 5). Seventy-two hours later, each group received fixed ratio (1:1) or combinations of fractions of the respective ED_50_ values of (9 + 3.1 mg kg^−1^), (4.5 + 1.6 mg kg^−1^), (2.25 + 0.8 mg kg^−1^), (1.125 + 0.4 mg kg^−1^), (0.6 + 0.2 mg kg^−1^), ED_50_ (XA/AQ), ED_50_ (XA/AQ)/2, ED_50_ (XA/AQ)/4, ED_50_ (XA/AQ)/8, and ED_50_ (XA/AQ)/16, respectively. Positive control (AL) and negative control (sham) mice received 1.14/6.9 mg kg^−1^ AL and 10 ml kg^−1^ vehicle, respectively.

#### Percentage chemo-suppression and parasitemia evaluation

Parasitaemia was tracked using thin blood smears made daily for 5 days by collecting three drops of blood from the tail of each mouse, fixed in absolute methanol, and stained in 10% Giemsa for 10 min to determine parasitaemia. The slides were microscopically examined at 100 × magnification. Parasitaemia was checked by counting infected red blood cells in hundred fields, divided by the total red blood cells in the hundred fields, and then multiplied by hundred and percentage parasitaemia calculated as follows:

$$\% Parasitaemia = \frac{Number\;of\;Plasmodium\; berghei - infected\;erythrocytes}{{Total\;number\;of\;erythrocytes}} \times 100.$$

Chemosupression or percentage inhibition of parasitaemia was computed by employing the following formula:

$$\% {\text{ inhibition}} = \frac{{\left( {Mean \;parasitaemia\;of\;negative\;control} \right) - \left( {Mean\;parasitaemia\;of\;test\;drug} \right)}}{Mean\;parasitaemia\;of\;negative \;control} \times 100.$$

### Data analysis

All statistical analyses were computed with the windows version of GraphPad Prism 7.0 (GraphPad Software, San Diego, CA, USA). Data were considered significant at *p* < 0.05 and were presented as the mean ± SEM. Tukey’s honest significant difference (HSD) test was used as a post hoc test. Two isobolograms which consisted of the ED_50_ of XA on the ordinate and ED_50_ of ART or AQ on the abscissa connected with a line of additivity were constructed. The ED_50_ of each drug was determined by linear regression analysis of the log dose–response curve (and a T-test was used for the comparison to a theoretical additive ED_50_ i.e. Z_add_). Z_add_ was computed with the following formulae:

$$Z_{add} = \left( f \right)\;ED50\;of\;ART + \left( {1 - f} \right)\;ED50\;of\;XA$$

and

$$Z_{add} = \left( f \right)\;ED50\;of\;AQ + \left( {1 - f} \right)\;ED50\;of\;XA,$$

where f is the fraction of each component in the mixture/ combination while the Var (variance) of Z_add_ was computed as follows:

$${\text{Variance}}\;{\text{of}}\;Z_{add} = \, f2 \, \left( {VarED50 \, of \, CYP} \right) \, + \, \left( {1 - f} \right) \, 2VarED50 \, of \, XA.$$

SEMs were calculated from these variances and fixed according to the drug’s ratio in the combination. If the effect of a drug combination was statistically different (ED_50_ significantly lower) and higher than the theoretically calculated equieffect of a drug combination in the same proportion, it has a supra-additive or synergistic effect.

## Results

### In vivo anti-malarial assay of xylopic acid, artesunate, and amodiaquine monotherapies

#### Effects of XA, ART, and AQ monotherapy on body weight

**Fig. 1:**
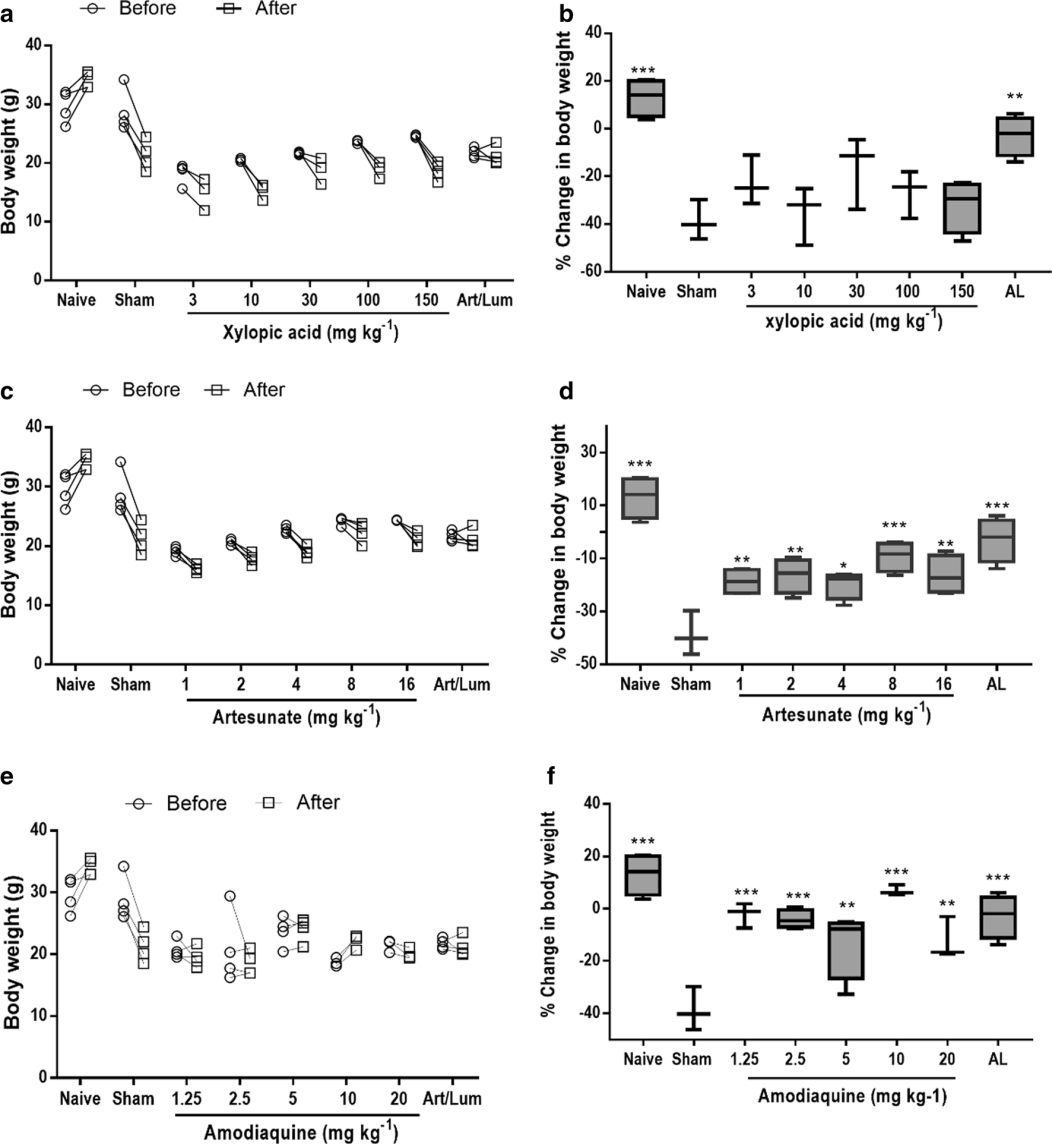
Bodyweight before infection and after treatment (left panel) and percentage change in body weight (right panel) for **a**, **b** xylopic acid, **c**, **d** artesunate, and **e**, **f** amodiaquine treated groups. Data are presented as mean ± SEM (n = 5), p < 0.05

Amodiaquine and artesunate treatment groups significantly reduced weight loss in mice infected with *P. berghei* (p = 0.001). In XA-treated groups, loss in body weight was not statistically significant. High doses of artesunate (8 mg/kg) and amodiaquine (10 mg/kg) showed an increase in body weight similar to the naïve and AL groups (Fig. 1).

#### Effects of XA, ART, and AQ monotherapy on post-treatment survival

**Fig. 2:**
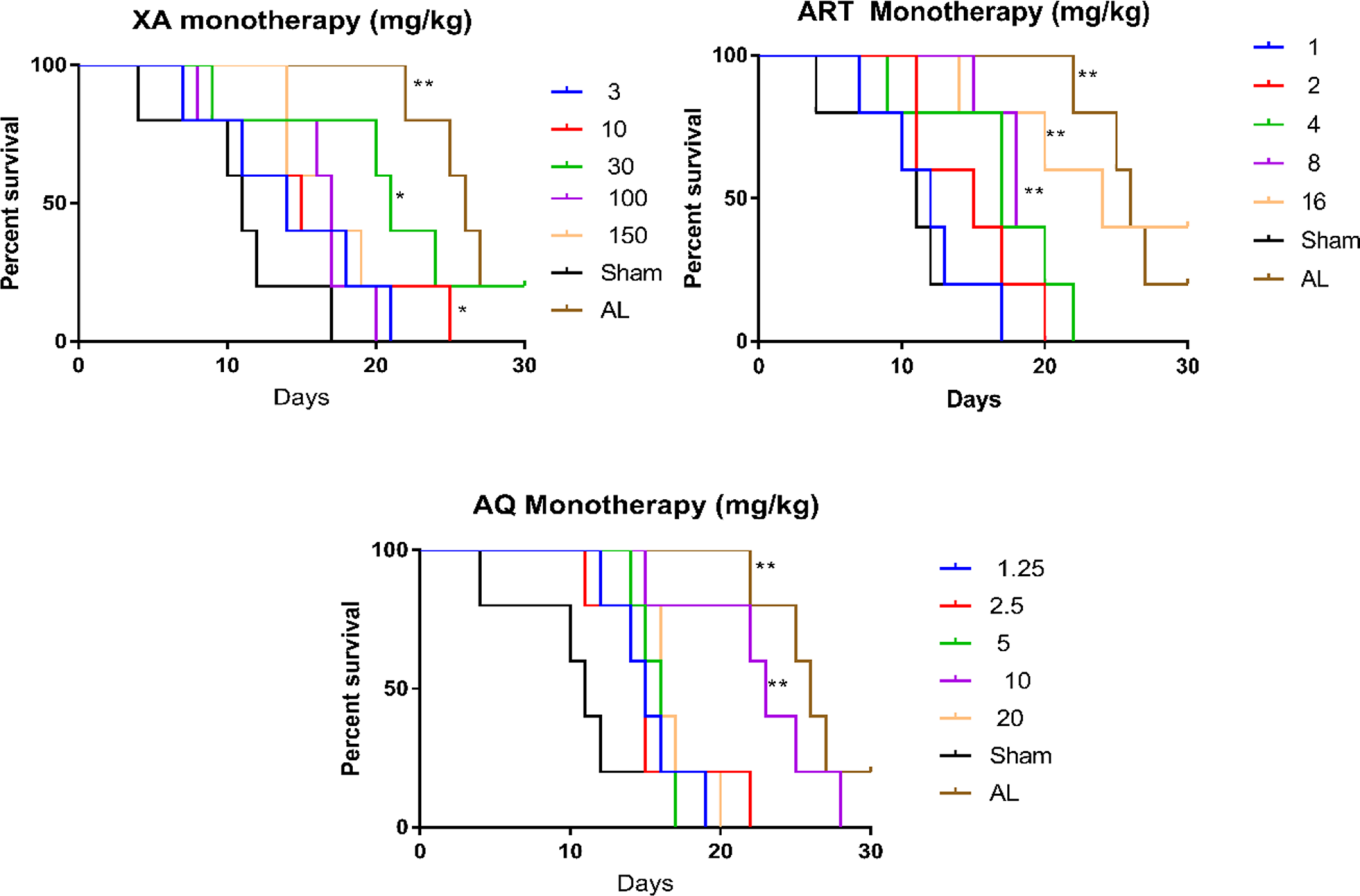
Kaplan–Meier survival curves comparing the survival of *P. berghei*-infected mice between sham and various treatment groups for 30 days post-infection. *p < 0.05, **p < 0.01

Artemether/lumefantrine-treated animals had 26 median survival days with a hazard ratio of 0.20 in the 30 days survival test (Fig. 2), representing the highest survival days and lowest hazard ratio in the treatment groups. Middle doses of XA (30 mg/kg), AQ (10 mg/kg), and high doses of ART-treated groups also significantly increased survival days in the 30 days survival test (p < 0.05) (Table 1, Fig. 2).

**Table 1:** 30-day survival analysis of *Plasmodium berghei*-infected mice after treatment with either xylopic acid, artesunate, amodiaquine, or artemether/lumefantrine

Treatment (mg/kg)	Median survival (days)	Hazard ratio (log-rank)	*p*-value
Naïve	30	–	–
Sham	11		
XA			
3	14	0.49	0.2017
10	15	0.49	0.2017
30	21	0.15	0.0269*
100	17	0.46	0.1401
150	17	0.32	0.0272*
ART			
1	12	0.84	0.7501
2	15	0.51	0.2088
4	17	0.39	0.0689
8	18	0.25	0.0064**
16	24	0.20	0.0064**
AQ			
1.25	15	0.47	0.1843
2.5	15	0.48	0.1842
5	16	0.53	0.2097
10	23	0.25	0.0064**
20	16	0.40	0.0812
AL	26	0.20	0.0018**

(n = 5), * means p < 0.05

### Anti-plasmodial effects of XA, ART, and AQ monotherapy

**Fig. 3:**
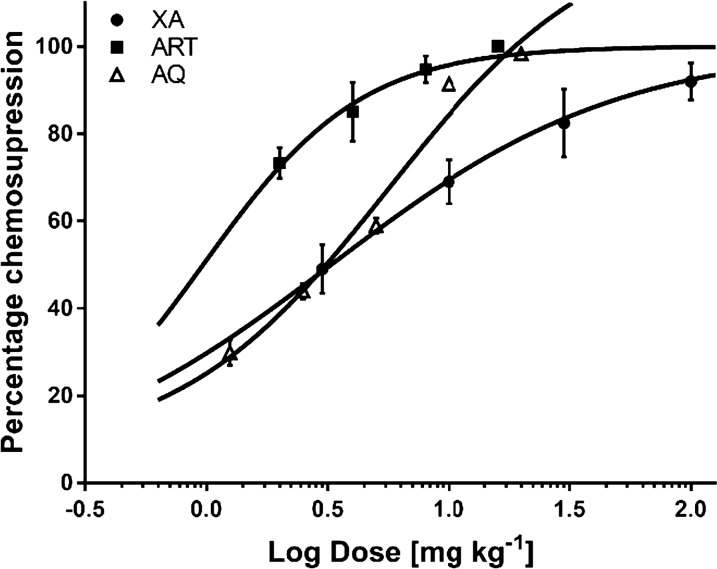
Log dose–response curves of percentage chemosupression in *P*. *berghei*-infected mice administered daily with xylopic acid, artesunate, or amodiaquine over 5 days. Data are presented as mean ± SEM (n = 5)

The anti-malarial activities of XA, ART, and AQ were individually evaluated in *P. berghei*-infected mice. Infection was established on day 3 (72 h post-infection) for all groups. ED_50_s for xylopic acid, artesunate, and amodiaquine were 9.0 ± 3.2, 1.61 ± 0.6, and 3.1 ± 0.8 mg/kg. By these results, the artesunate was 1.9 times more potent than amodiaquine, and amodiaquine was 2.9 times more potent than xylopic acid (Fig. 3).

### In vivo* assessment of xylopic acid-artesunate and xylopic acid-amodiaquine co-administration of PbA-induced malaria*

#### Effects of XA + ART and XA + AQ combination on weight loss

**Fig. 4:**
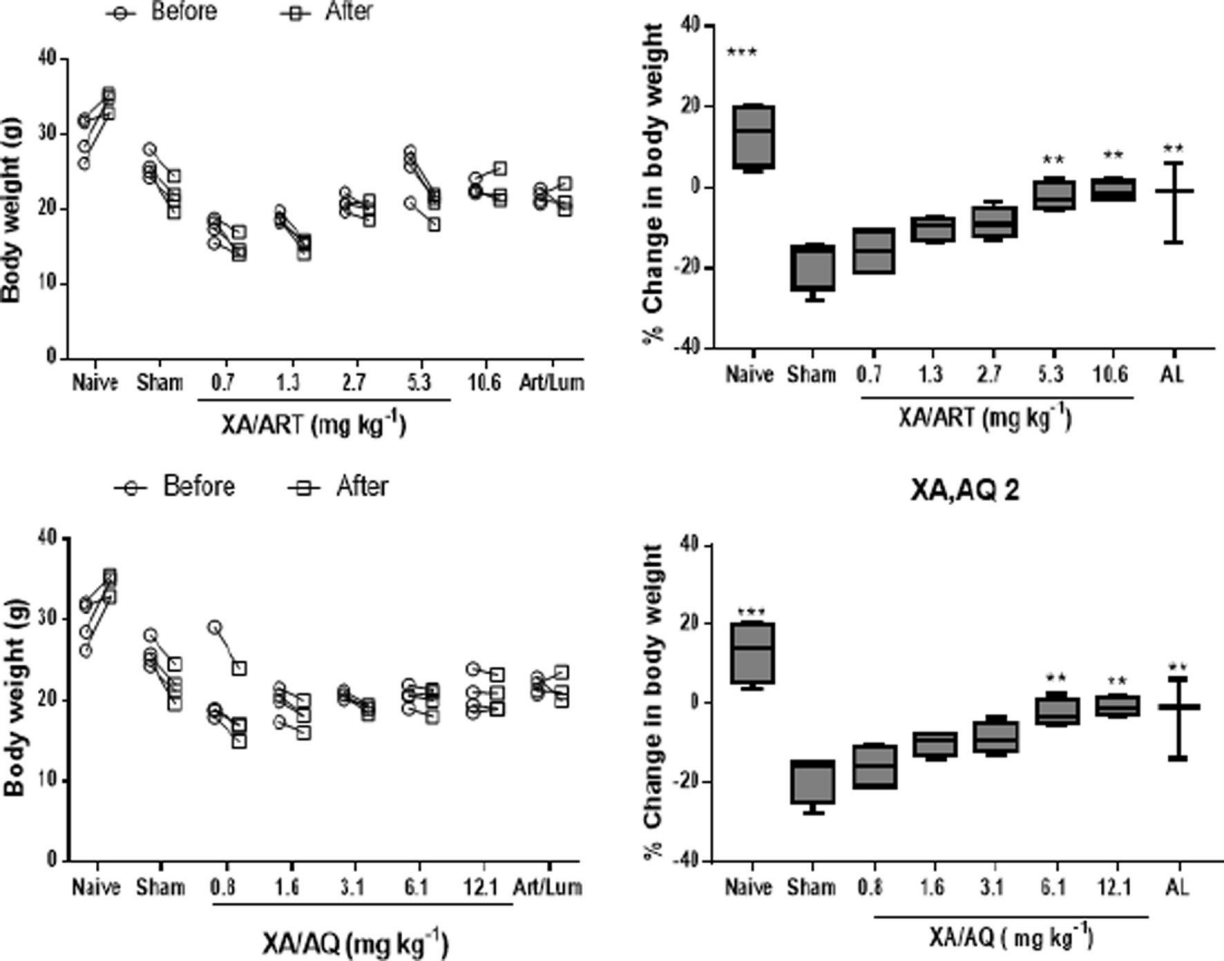
Bodyweight before infection and after treatment and percentage change in body weight for **a**, **b** xylopic acid-artesunate, and **c**, **d** xylopic acid-amodiaquine combination-treated groups. Data are represented as mean ± SEM (n = 5), *p* < 0.05

AL, 5.3 mg/kg XA + ART, 10.6 mg/kg XA + ART, 6.1 mg/kg XA + AQ, and 12.1 XA + AQ treated groups significantly reduced loss in body weight compared to the sham-treated group (p < 0.05) (Fig. 4).

### Effects of XA + ART and XA + AQ combination on parasetemia and chemosuppression

**Fig. 5:**
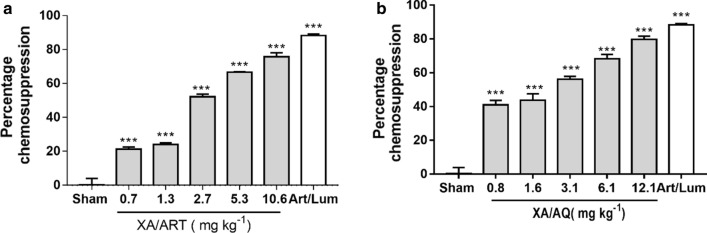
Percentage chemosupression for **a** xylopic acid-artesunate and **b** xylopic acid-amodiaquine treated *P. berghei*-infected mice. Data are presented as mean ± SEM, (n = 5), *p* < 0.05

**Fig. 6:**
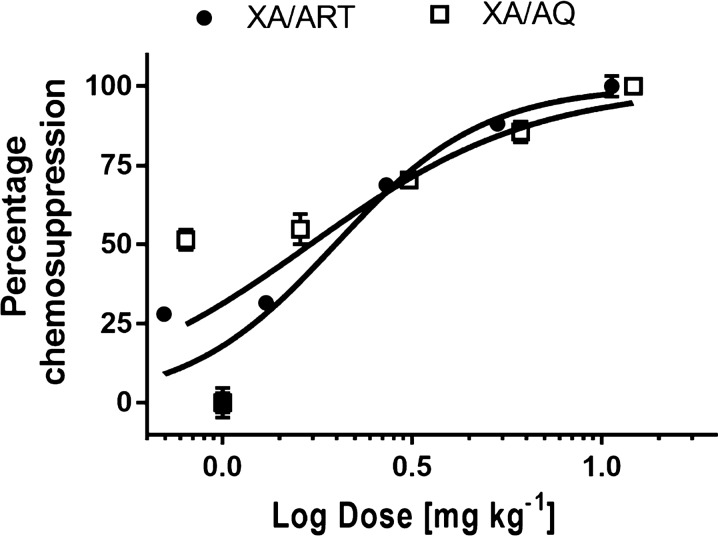
Log dose–response curves for *P*. *berghei*-infected mice treated daily with xylopic acid/artesunate (XA + ART) combination, and xylopic acid-amodiaquine (XA + AQ) combination over 5 days. Data are presented as mean ± SEM (n = 5)

Percentage chemosupression for the highest combination doses of both XA/ART (Table 2) and XA/AQ (Table 3) suppressed parasite growth similar to artemether/lumefantrine (Fig. 5). The ED_50_s for xylopic acid-artesunate and xylopic acid-amodiaquine were 1.98 ± 0.33 and 1.69 ± 0.83, respectively (Fig. 6).

**Table 2:** Percentage parasitemia and chemosupression of mice treated with different doses of xylopic acid + artesunate combination for 5 days

Treatment (ED_50_ mg/kg)	Dose (mg/kg)	% Chem supp Day 6
Sham	0.50 ml	–
(XA/ART)16	0.6:0.1	21.5 ± 0.6
(XA/ART)/8	1.13:0.2	24.1 ± 0.5
(XA/ART)/4	2.25:0.4	52.2 ± 0.8
(XA/ART)/2	4.5:0.8	66.8 ± 0.1
(XA/ART) 10.6	9.0:1.61	75.7 ± 1.2
AL 1.14	–	84.6 ± 1.6

Data are presented as mean ± SEM, (n = 5)

**Table 3:** Percentage parasitemia and chemosupression of mice treated with different doses of xylopic acid + amodiaquine combination for 5 days

Treatment (ED_50_ mg/kg)	Dose (mg/kg)	% Chem sup Day 6
Sham	0.50 ml	–
(XA/AQ)/16	0.6:0.2	41.2 ± 1.3
(XA/AQ)/8	1.125:0.4	43.9 ± 1.9
(XA/AQ)/4	2.25:0.8	56.4 ± 0.8
(XA/AQ)/2	4.5:1.6	68.4 ± 1.3
(XA/AQ) 12.1	9.0:3.1	79.8 ± 0.9
AL 1.14	–	88.3 ± 0.4

Data are presented as mean ± SEM, (n = 5)

### Survival analysis for combination therapy

**Fig. 7:**
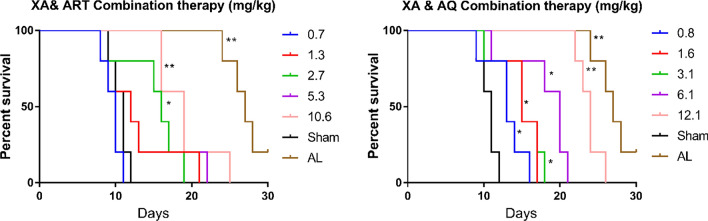
Kaplan–Meier survival curves comparing the 30 days-post treatment survival of *P. berghei*-infected mice treated with XA/ART, XA/AQ, or AL

The XA/AQ combination doses significantly delayed death in in *P. berghei*-infected mice in a 30-day survival test, similar to the AL treated group. Likewise, the high doses of the XA/ART combination increased the survival days of the *P. berghei*-infected mice (Table 4, Fig. 7).

**Table 4:** Thirty-day survival analysis of *Plasmodium berghei*-infected mice after treatment with xylopic acid and amodiaquine, and xylopic acid and artesunate

Treatment (mg/kg)	Median survival (days)	Hazard ratio (log-rank)	*p*-value
Sham	10.5		
(XA/ART)			
0.7	10	0.52	0.1766
1.3	12	0.59	0.2941
2.7	16	0.35	0.0374*
5.3	16	0.35	0.0374*
10.6	19	0.23	0.0021**
(XA/AQ)			
0.8	13	0.34	0.0297*
1.6	15	0.14	0.0297*
3.1	15	0.33	0.0224*
6.1	20	0.29	0.0112*
12.1	24	0.23	0.0021**
AL	27	0.21	0.0021**

(n = 5), * means p < 0.05

#### Isobolographic analysis of antiplasmodial effects of XA and ART, and XA and AQ co-administration

**Fig. 8:**
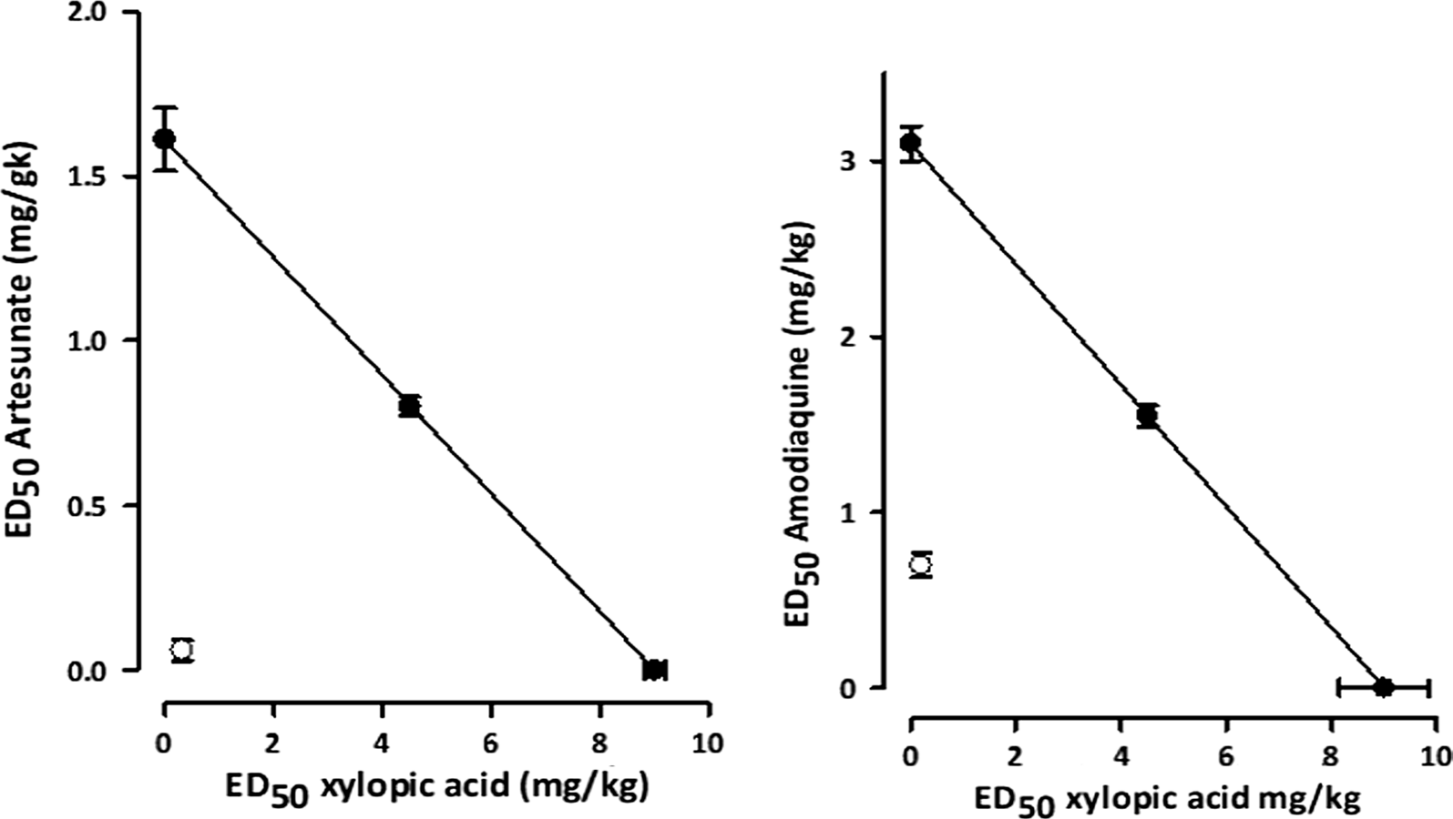
Isobologram of the co-administration of xylopic acid and artesunate, and xylopic acid and amodiaquine. Filled circles show theoretical ED_50_ ± SEM, while open circles show experimental ED_50_ ± SEM. The line of additivity connects the ED_50_ of xylopic acid on the abscissa to that of artesunate and amodiaquine on the ordinate

Xylopic acid-artesunate co-administration had a theoretical ED_50_ (Z_add_) of 5.3 ± 2.61, whereas the experimental ED_50_ (Z_exp_) was obtained as 1.98 ± 0.25 (Table 5). Also, the co-administration of xylopic acid and amodiaquine had a theoretical ED_50_ value of 6.05 ± 2.0; however, the experimental ED_50_ was 1.69 ± 0.42. Thus, the Z_exp_ for both combinations lies significantly below the line of additivity since the interaction index was calculated to be 0.37 and 0.28 for xylopic acid-artesunate and xylopic acid-amodiaquine co-administration, respectively (Fig. 8).

**Table 5:** Theoretical (Z_add_), and experimental (Z_exp_) ED_50_ of xylopic acid and artesunate, and xylopic acid and amodiaquine co-administration in the anti-malarial assay

ED_50_s (XA/ART 1:1)	Anti-malarial activity	ED_50_s (XA/AQ 1:1)	Anti-malarial activity
Z_add_ (mg/kg)	5.3 ± 2.61	Z_add_ (mg kg^−1^)	6.05 ± 2.0
Z_exp_ (mg/kg)	1.98 ± 0.25	Z_exp_ (mg kg^−1^)	1.69 ± 0.42
Interaction index	0.37	Interaction index	0.28

Data are presented as mean ± SEM

## Discussion

*Plasmodium falciparum* has developed resistance to antiplasmodial agents over the years and has been reported to acquire resistance to currently used anti-malarial drugs [22]. Growing evidence of the resistance of *P. falciparum* to even artemisinin derivatives calls for the urgent need for more efficient and safer anti-malarials and nature remains a key source for such novel anti-malarial agents [23]. Combination therapy is a good strategy in antimicrobial chemotherapy because it enhances the probability of sustained efficacy in the advent of parasite resistance to one agent [24]. The combination also helps in preventing the development of resistance due to their multiple mechanisms of action making evasion by the parasite significantly difficult. Combination therapy also improves efficacy when the agents act synergistically [9]. Against this background, this study examined the effectiveness of combining each of two established anti-malarial agents, artesunate and amodiaquine, with an investigational antiplasmodial agent, xylopic acid.

Xylopic acid, extracted from the unripe fruits of *Xylopia aethiopica* has been examined previously to have antiplasmodial, anti-inflammatory, antipyretic [13], and analgesic [17] properties. Also, it has been recently reported to act synergistically when combined with other plant-derived antiplasmodial compounds such as cryptolepine [14]. These properties are crucial in the management of malaria symptomatology, making xylopic acid a potential anti-malarial agent for further drug development and a good candidate for combination therapy in anti-malarial chemotherapy.

Combining xylopic acid with either artesunate or amodiaquine showed a remarkable suppression in parasite growth similar to the artemether/lumefantrine. Although, monotherapy of XA, ART, and AQ also suppressed parasite growth compared to artemether/lumefantrine it occurred at higher doses. An isobolographic analysis was employed to determine the enhanced or improved potency and efficacy of xylopic acid-artesunate, and xylopic acid-amodiaquine combination therapies. An isobolographic analysis gives a central basis for evaluating whether a biological response induced by a mixture of agents is smaller, equal, or greater on the concept of dose additivity and the basis of the components or agents’ activities [25]. The co-administration of xylopic acid and artesunate showed significant antiplasmodial activity in comparison to the sham-treated mice. The isobologram showed that when xylopic acid and artesunate are administered together, the Z_exp_ was significantly below the line of additivity (“additive” isobole) and the Z_add_, which means the two drugs have a synergistic anti-plasmodial effect. The interaction index of 0.37, which is significantly less than 1, confirms a synergistic relationship [26] and a supra-additive effect between artesunate and xylopic acid.

Compared to a recent study by Ameyaw et al. [14], combining xylopic acid and artesunate gave a higher supra-additivity and synergistic interaction than xylopic acid and cryptolepine combination, probably, due to the high synergistic property of artesunate [27–29]. Nevertheless, xylopic acid-cryptolepine co-administration showed a higher parasite clearance rate of 78% for the higher dose combination compared to the 75% for the higher dose combination of xylopic acid and artesunate. Another study that examined the chemotherapeutic interactions between anti-malarial drugs and antiretroviral drugs observed an increase in antimalaria activity when ART was combined with lopinavir/ritonavir (LR) on day 5 post-infection in mice infected with *P. berghei* [15] confirming the synergistic interaction of artesunate with other potent drugs.

The observed increased antiplasmodial activity of the XA/ART combination could also be attributed to the two drugs interacting with several targets in the parasite. XA inhibits plasmodium dehydrogenase [30], an enzyme that catalyzes the reduction of pyruvate to lactate, crucial for energy production, whilst artemisinin derivatives are believed to undergo reductive activation of the peroxide group in the presence of ferrous ion which is released upon haemoglobin digestion within the food vacuole of the parasite [14, 31]. This forms a carbon-centered radical which alkylates vital parasite proteins such as heme and membrane-associated parasite proteins [32, 33]. Thus, the inhibition of different metabolic steps in *Plasmodium* haemoglobin digestion of parasite glycolysis might contribute to the enhanced antiplasmodial activity of ART and XA.

Furthermore, the anti-inflammatory properties of xylopic acid may have contributed to the limiting survival of the parasite. Osafo and colleagues recently reported the anti-inflammatory properties of xylopic acid against various phlogistic agents (bradykinin, serotonin, carrageenan, histamine, and prostaglandin E_2_). XA inhibited albumin denaturation, and also maximal edema, and average paw thickness induced by the phlogistic agents for both prophylactic and therapeutic studies. It also inhibited the arachidonic acid pathway [34, 35]. Inflammation plays a key role in the pathogenesis of malaria. Following *P. berghei* infection, splenic dendritic cells, CD8α^+^ and Clec9A^+^ phagocytose, and cross-present parasite antigens which lead to the priming of parasite-specific CD4^+^ and CD8^+^ T cells. Circulating parasitized red blood cells (pRBC) adhere to the endothelium of blood vessels releasing inflammatory ligands such as hemozoin crystals which contain parasite DNA. These stimuli are responded to by the release of cytokines and chemokines leading to the upregulation of adhesion molecules (ICAM, VCAM) and receptors (CXCR3) capable of presenting antigens [36]. When adhesion molecules are upregulated, they aid in the primary rolling and tethering interactions between lymphocytes, granulocytes, and monocytes to endothelial cells at sites of tissue injury. If perturbed endothelial cells interact with monocytes along with synergistic action of proinflammatory molecules, they potentially exacerbate tissue factor expression and subsequently activate endothelial cells sustaining coagulation-inflammation cycle [37–40], hence, promoting the “vicious” cycle of coagulation-inflammation of sepsis, which is found to be crucial in malaria pathogenesis. Also, the adherence of parasites to the endothelium with the help of upregulated adhesion molecules following inflammation helps in the survival of parasites. Hence, the acute anti-inflammatory properties might prevent the coagulation-inflammation cycle contributing to the limited growth and survival of mice treated with xylopic acid-amodiaquine, and xylopic acid-artesunate combination.

*Plasmodium* parasites have over the years evolved several biomolecular strategies for escaping immune response to secure parasite survival in the host. One-way parasites achieve immune escape is via the exploitation of host components such as inflammation and platelets that can cause infected red blood cells (iRBCs) and uninfected RBCs to agglutinate promoting the appropriate microenvironment for sequestration [41–43]. The release of a collection of mediators of inflammation may either result in an exacerbated immune response leading to pathology [44]. CD4^+^ T-helper cells have been reported to be involved in malaria conferring protection. However, they have also been implicated in immune evasion and malaria pathogenesis [45]. Despite all this, the demonstrated significant anti-inflammatory properties of XA [34, 46] might have prevented the poor outcome of malaria in the XA-ART, XA-AQ treated groups.

A combination of xylopic acid and amodiaquine showed enhanced activity due to their synergistic interaction. Like the XA/ART combination, XA/AQ interaction also showed an interaction index of 0.13, which is significantly different from [1]. XA/AQ isobologram lay below the line of additivity, confirming the synergistic interaction between the two compounds. The precise molecular mechanisms by which these two agents act is not very clear, but several proteins in the parasite might be a target. AQ metabolite (desethylamodiaquine) is thought to accumulate in parasites food vacuole preventing the conversion of toxic haem produced due to intraerythrocytic parasite digestion of haemoglobin into crystalline haemozoin which is non-toxic to the host but irreversibly toxic to the parasite as a result of the build-up of haem levels [33]. Previous works on anti-malarial combination therapies have shown that, when aspartyl PI is combined with other haemoglobin digestion inhibitors, it acts synergistically [33] but acts antagonistically with vacuole plasmepsin inhibitors [47]. The mechanisms employed by individual drugs of the combination to inhibit metabolic steps in the digestion of haemoglobin may result in the enhanced anti-malarial activity of XA in the presence of AQ and ART shown in this study.

In malaria treatment, like any other infectious disease, it is crucial not only to pay attention to the pathogen but also the reduction of the symptoms of the infection which independently increases the pathogen burden [48]. Among the several general features of malaria infection is the loss of body weight. Weight loss can be attributed to metabolic function disturbance and hypoglycemia caused by malaria parasite infection [49–51]. Hypoglycaemia in malaria patients can also be attributed to the increase in glucose uptake by the febrile host and the parasite. Alternatively, the host’s glucose production may be impaired [52]. Thus, an ideal anti-malarial drug is anticipated to prevent the decrease in body weight of mice due to rising parasitaemia, which is crucial for mice survival. AQ and ART prevented the loss of weight of infected mice significantly (p = 0.001). Although the XA monotherapy experiment did not significantly prevent weight loss, the combination therapy with ART and AQ showed a significant reduction in weight loss in the 10.6 mg/kg and 12.1 mg/kg combination doses. This observation correlates with other studies where a combination of xylopic acid and cryptolepine prevented a loss in body weight in mice infected with *P. berghei* [14, 18]. It is possible that the enhanced antiplasmodial effect of the combination therapy suppressed parasite growth which led to a decrease in glucose intake by the parasite and also restored the animals’ appetite as they recovered from the disease.

All the characteristics of an ideal anti-malarial agent should be able to prevent eventual death caused by parasites by suppressing the growth of parasites, thereby reducing the risk of death. An increase in parasite growth causes various symptoms of malaria which eventually leads to the death of the hosts [53]. The hazard ratio is used in drug treatment to describe the relative risk of complication when compared to event rates. In this study, the hazard ratio was measured to describe the outcome of the drug’s safety in the malaria treatment in relation to mice survival days. The XA and AQ monotherapy showed a significant increase in the survival days for the middle doses while the high doses showed increased parasite clearance but reduced median survival days and increased hazard ratios. Notwithstanding, the high doses of the ART-treated group showed significant increased median survival days and reduced hazard ratio similar to AL. Surprisingly, in the combination therapy, the XA/ART treatment groups showed higher parasite clearance compared to XA/AQ, but their median survival day was only significant in the high doses with a mean hazard ratio of 0.40, meanwhile, XA and AQ which showed significant increased survival days and reduced hazard ratio in only the middle doses during the monotherapy, had a significant increase in survival days for all the combination doses with a mean hazard ratio of 0.27 similar to AL. It is a possibility that the early death of the animals in the XA/ART could have been due to the toxicity of the combination since there was high parasite clearance [14, 54]. AQ has been consistently reported to be relatively toxic [55, 56]. Several studies indicate amodiaquine combination therapy could cause fetal death in animals, and indeed, there have been reports of fetal resorption in early pregnancies [57]. The WHO, hence, recommends the avoidance of these drugs in the first trimester, but the problem can still exist if some women fail to recognize their conception at early stages. Notwithstanding, there was increased survival days for the xylopic acid-amodiaquine treated group in relation to the xylopic acid-artesunate treated groups, although, it had a lower parasite clearance. Thus, hypothetically, the combination of xylopic acid with AQ reduced the toxicity of AQ. Median survival for both AL and XA/AQ was statistically significant.

## Conclusion

The findings of this study is heartwarming in the light of the report of growing resistance to current artemisinin [58]. The combination of xylopic acid with either amodiaquine or artesunate seemed to have increased efficacy since lower doses of each of the agents were required to produce a significant therapeutic effect, and also reduced toxicity in the xylopic acid and amodiaquine combination.

## Data Availability

The data are available only upon request from the authors.

## Abbreviations

ACT: Artemisinin-based combination therapy
ART: Artesunate
AQ: Amodiaquine
CPE: Cryptolepine
DCTs: Drug combination therapies
ED_50_: Median effective dose
HSD: Honest significant difference
ICR: Institute for cancer research
LPS: Lipopolysaccharide
pRBC: Parasitized red blood cells
SEM: Standard error of mean
SP: Sulfadoxine-pyrimethamine
XA: Xylopic acid
Z_add_: Theoretical ED_50_
Z_exp_: Experimental ED_50_
